# Direct observation of charge state in the quasi-one-dimensional conductor Li_0.9_Mo_6_O_17_

**DOI:** 10.1038/srep20721

**Published:** 2016-02-08

**Authors:** Guoqing Wu, Xiao-shan Ye, Xianghua Zeng, Bing Wu, W. G. Clark

**Affiliations:** 1 College of Physics Science and Technology, Yangzhou University, Yangzhou 225002, China; 2 Department of Physics, University of West Florida, Pensacola 32514, USA; 3 Department of Math and Computer Science, Fayetteville State University, Fayetteville, North Carolina 28301, USA; 4 Department of Physics and Astronomy, University of California, Los Angeles, California 90095, USA

## Abstract

The quasi-one-dimensional conductor Li_0.9_Mo_6_O_17_ has been of great interest because of its unusual properties. It has a conducting phase with properties different from a simple Fermi liquid, a poorly understood “insulating” phase as indicated by a metal-“insulator” crossover (a mystery for over 30 years), and a superconducting phase which may involve spin triplet Cooper pairs as a three-dimensional (*p*-wave) non-conventional superconductor. Recent evidence suggests a density wave (DW) gapping regarding the metal-“insulator” crossover. However, the nature of the DW, such as whether it is due to the change in the charge state or spin state, and its relationship to the dimensional crossover and to the spin triplet superconductivity, remains elusive. Here by performing ^7^Li-/^95^Mo-nuclear magnetic resonance (NMR) spectroscopy, we directly observed the charge state which shows no signature of change in the electric field gradient (nuclear quadrupolar frequency) or in the distribution of it, thus providing direct experimental evidences demonstrating that the long mysterious metal-“insulator” crossover is not due to the charge density wave (CDW) that was thought, and the nature of the DW gapping is not CDW. This discovery opens a parallel path to the study of the electron spin state and its possible connections to other unusual properties.

The physical properties of quasi-low-dimensional (Q1D) conductors have been the subject of numerous investigations since these materials allow many fundamental theories of one- and two-dimensional systems to be directly tested^1^,^2^,^3^,^4^. It has been found^4^,^5^ that one-dimensional (1D) electron gas is not stable at low temperatures and correlation effects (interactions/fluctuations among the electron charges and/or spins) can lead to a rich variety of phase transitions and to different collective modes of condensate phase excitations. Depending on the details^6^,^7^ of the electron-electron and/or electron-phonon interactions, various quantum ground states, such as CDW, spin-density wave (SDW), and singlet (*s*-wave or *d*-wave) or triplet (*p*-wave) superconductivity may occur. Among which, CDW or SDW appears to be a precursor which sets the stage for superconductivity^4^,^5^,^8^. These nature phenomena have been observed^1^,^2^,^3^,^4^,^6^,^7^ in a number of low dimensional organic and non-organic conductors including cuprate and Fe-based high-*T*_c_ superconductors where the Fermi surface is highly anisotropic, suggesting a peculiarity of their electron charge and/or spin state associated with the formation of each of these long-range ordered phases.

In this paper we present an nuclear magnetic resonance (NMR) study of the low temperature metal-“insulator” crossover^9^,^10^,^11^,^12^,^13^,^14^, which is one of the most mysterious properties^5^,^14^,^15^,^16^,^17^ of the Q1D paramagnetic conductor lithium purple bronze, Li_0.9_Mo_6_O_17_. Even though intensive experimental studies have been performed since 1980s, the mechanism of this crossover remains unsolved, while four completely different mechanisms^9^,^10^,^11^,^12^,^13^,^14^,^15^,^17^,^18^,^19^ have been theoretically proposed: CDW, SDW, localization due to disorder (Anderson type), and Luttinger liquid. When the applied magnetic field *B*_0_ = 0, the crossover appears at temperature *T*_MI_ = 24 K, while when *B*_0_ ≠ 0 this temperature can shift somewhat depending on the direction of it^13^,^19^. As for the superconductivity, the transition is at temperature *T*_c_ = 2.2 K^8^,^9^,^20^,^21^, and the superconductivity has been found to be three dimensional (3D)^20^,^21^,^22^,^23^. Thus this indicates that there is also an electron Q1D to 3D dimensional crossover. According to the thermal expansion data^8^, here the Q1D to 3D dimensional crossover of the conducting electrons is also found to take place gradually starting at or near the metal-“insulator” crossover temperature.

Most recent thermopower measurements show extreme thermoelectric effect anisotropy^24^,^25^, and theoretical studies^22^,^23^ suggest that when *B*_0_ is applied perpendicular to the lattice *b*-axis (*B*_0_ ⊥ *b*) and along the *c*-axis (in the sample *bc*-plane), there is a re-entrant superconductivity at high fields. These studies agree with those of the resistivity measurements in the applied magnetic field^20^,^21^, in which the metal-“insulator” crossover is viewed as the evidence of a DW gap (either CDW or SDW) formation^20^,^21^,^26^. They may shed new light on the understanding of the unknown properties, as they imply the significance of the electron charge and/or spin state, as well as its possible changes, associated with the metal-“insulator” crossover and its possible connection to the dimensional crossover as well as to the 3D superconductivity^20^,^21^,^22^,^23^. However, controversy exists^27^ in view of some of the same sets of experimental data presented previously, and also the authors with the μsR data^28^ argue that SDW is not supported. Moreover, direct electron charge and/or spin state evidences, which are key important toward resolving the mysteries, have not been reported.

Here we provide a direct observation of the electron charge state for the low temperature metal-“insulator” crossover phenomenon, with our detailed temperature, field and angular dependences of the ^7^Li-/^95^Mo-NMR spectroscopy measurements on a single crystal of Li_0.9_Mo_6_O_17_. It is well-known that NMR is a versatile local probe capable of directly measuring the local electric and magnetic field including the electron charge and spin statics & dynamics at the atomic scale. Here we focus on the electron charge dynamics & statics surrounding the ^7^Li and ^95^Mo nucleus, which serve as the direct probes for the observation, with the experimental data that has the contributions from all types of sources including the lattice (electron-phonon coupling)^6^ and non-lattice contributions (such as the argument of possible electron-electron repulsion as that in the regime of a Luttinger liquid theory, a purely electronic origin)^8^. Our measurement has a sensitivity of 0.01 kHz in frequency, which allows us to be able to detect the local electric and/or magnetic field changes in the order of 10^2^–10^3^ times smaller than the known values previously reported in other Q1D materials^29^,^30^, associated with a possible CDW or SDW formation, or if any other state change occurs.

Figure 1a is the schematic of the experimental set-up with sample rotations around the lattice *b*-axis in the applied field *B*_0_ used in our NMR measurements, where the angle *θ* = 0^o^ corresponds to *B*_0_ parallel to the *a*-axis (*B*_0_ || *a*). As we know, Li_0.9_Mo_6_O_17_ has a highly anisotropic conductivity^20^,^21^ of 250:10:1 along the lattice *b*, *a* and *c* axes, respectively (i.e., *b* is the conducting axis), with a monoclinic (space group *P*2_1_/*m*) crystal structure^28^, as shown in Fig. 1b. More experimental details are described in the Methods section.

Figure 2a shows the ^7^Li-NMR spectra at a typical temperature *T* = 275 K with *B*_0_ = 9 T, at various angles, plotted as the ^7^Li-NMR free-induction decay (FID) absorption amplitude versus the MMR frequency shift *ν* − *ν*_*L*_, where *ν* is the NMR resonance frequency of the ^7^Li nucleus. Here *ν*_*L*_ is called the Larmor frequency, a constant determined by the value of *B*_0_ and the gyromagnetic ratio of ^7^Li. As expected theoretically^29^, the ^7^Li-NMR spectrum has a central line (P_C_) plus two symmetric quadrupolar satellites (P_S1_ and P_S2_), due to the ^7^Li spin quantum *m* = +1/2 ↔ −1/2 (central) and ±3/2 ↔ ±1/2 (satellites) transitions, respectively, as a spin *I* = 3/2 nucleus. Only three NMR lines are observed because all the Li sites are equivalent due to the space group *P*2_1_/*m* symmetry.

Noticeably, as the angle *θ* varies, the satellites P_S1_ and P_S2_ exchange their positions across the angle *θ* = 54.7° (called “magic angle”)^31^,^32^. Their frequency shifts (*ν* − *ν*_*L*_ ≡ *ν*_S_) have a rather strong angular dependence, which is in sharp contrast to that of the central line P_C_. This is because, generally, NMR spectrum satellites and central line have different origins: the central line is magnetic, while the satellites are quadrupolar – because of the quadrupolar interaction of the probe nucleus’s quadrupole moment (*Q*) with the electric field gradient (EFG) at the probe nucleus (under the high field limit)^29^. The EFG comes from the surrounding charges at all the lattice sites (called contribution of ligand lattice), plus electron orbital overlaps and charge covalence, according to the well-known point-charge model^31^,^32^. The quadrupolar interaction contribution to the satellites is dominant as it is in the first order, while to the central line is in the second order and thus usually negligible. Therefore, an NMR spectrum satellite of a probe nucleus can be used as a direct probe for the observation of the electron charge state, an intrinsic electronic behavior.

Figure 2b shows the angular dependence of the ^7^Li-NMR spectrum frequency shifts at various temperatures, from which we obtained the experimental value of ^7^Li quadrupolar frequency *ν*_Q_, a measure^31^,^32^ of the EFG tensor (*V*_zz_), *ν*_Q_ ≈ 44 kHz (detailed analysis can be found in the Supplementary Information). These data also indicate a highly symmetric electric field environment, where the z-component (*p*_z_) of the EFG principle axes is found to be ⊥ *b* and along the lattice *a*-axis at the Li site. But it shows no signature of change in the value of *ν*_Q_ (or EFG), upon cooling over a wide range of temperatures (including the crossover temperature at ~24 K).

In order to examine possible field effect on the observed EFG, we varied the magnitude of the magnetic field *B*_0_. This is shown in Fig. 3a, plotted as ^7^Li-NMR quadrupolar split Δν_s_ (Δν_s_ ≡ ν_s1_−ν_s2_) versus angle *θ* with *B*_0_ = 12 T, where *ν*_s1_ and ν_s2_ are the frequency shifts of the satellites P_S1_ and P_S2_, respectively (see Fig. 2b). For comparison, the data with *B*_0_ = 9 T at various temperatures are also displayed. No magnetic field dependence on the value of *ν*_Q_ (or EFG) is observed, which is also understandable since the satellites have a non-magnetic origin.

To further consolidate this observation, we performed similar NMR measurements at *B*_0_ = 14.8 T with the ^95^Mo nucleus (spin *I* = 5/2). The data are shown in Fig. 3b, where the values of ν_s1_ and ν_s2_ are the frequency shifts of the two inner NMR spectrum satellites (*m* − 1/2 = ±1), and ν_s3_ and ν_s4_ are the two outer ones (*m* − 1/2 = ± 2) right next to the inner satellites. With the same analysis used above for the ^7^Li, we obtained the ^95^Mo quadrupolar frequency *ν*_Q_ ≈ 65 kHz. The data also show a highly symmetric electric field environment, except that the z-component (*p*_z_) of the EFG principle axis at the Mo sites is along the lattice *c*-axis (note, the conduction electrons come from the Mo atoms). There is no signature of change in the value of *ν*_Q_ (or EFG) at the ^95^Mo, either, upon cooling in temperature. These results are summarized in Fig. 4, together with those of the ^7^Li nucleus, including the distribution of *ν*_Q_ as a function of temperature (300–2 K) and/or magnetic field (2.7–14.8 T) (see discussions in the Supplemental Information).

Finally, the values of *ν*_Q_ (EFG) obtained above with both ^7^Li and ^95^Mo nuclei can be theoretically calculated using the point-charge model^29^,^30^. Our theoretical estimation (detailed in the Supplemental Information) indicates that on the average at both nuclei, 1) the charge covalence contribution to *ν*_Q_ (EFG) has a similar magnitude as that from the ligand lattice, while the contribution of the orbital overlap is negligible, and 2) among the ligand lattice the charges from the Mo electrons have a contribution ~1.5 times larger than that of the charges from the oxygen. This indicates the effectiveness of the observation probes using ^7^Li and/or ^95^Mo nuclei.

In summary, we presented a direct observation of the electron charge state in Li_0.9_Mo_6_O_17_. The parameters of the EFG (nuclear quadrupolar frequency) are found by our ^7^Li- and ^95^Mo-NMR measurements and also theoretically estimated. We showed no sign of change in the EFG or in the distribution of it at the atomic scale, as a function of temperature and/or applied magnetic field, i.e., there is no possibility for a lattice-driven or a purely electronic CDW. Thus, we provided a direct experimental evidence demonstrating that the long mysterious metal-“insulator” crossover is not due to the CDW, and the nature of the observed DW gapping is not a CDW. Our discovery lays the foundation for the understanding of the unusual properties of Li_0.9_Mo_6_O_17_, and opens a parallel path to the study of the electron spin state at the metal-“insulator” crossover and of its potential connection to the electron dimensional crossover as well as to the spin triplet superconductivity in low dimensional electron systems in general.

## Methods

High quality single crystals of Li_0.9_Mo_6_O_17_ were grown using a temperature-gradient flux method^8^,^10^. The sample used for the measurement has a length ~1.7 mm and a width ~1.0 mm, while the thickness at one end is 0.3 mm and at the other end is ~0.6 mm. The sample mass is 1.5 mg. The NMR coil was made from 50 μm diameter copper wire wound with ~30 turns. The ^7^Li-NMR experiments were conducted with a spectrometer and probe built at UCLA Clarklab (W. G. Clark), and the ^95^Mo-NMR measurements on the same sample were performed at the Grenoble High Magnetic Field Laboratory, France with field *B*_0_ = 14.8 T. The sample was fixed on the goniometer in the NMR probe during the measurements so that it can rotate around the lattice *b*-axis.

The “smash tickle” method developed^33^ by Clark *et al.* was used for the ^7^Li-NMR measurements, with our consideration that the ^7^Li spin-lattice relaxation time goes extremely long at low temperatures. The ^95^Mo-NMR measurements used standard spin-echo techniques^31^,^32^, with number of averages up to 10,000 for the signal recording, due to the very small gyromagnetic ratio and the very small natural abundance of the ^95^Mo nucleus. Thus, noticeably, these are extremely difficult experiments.

The calibration of each applied magnetic field *B*_0_ used for the ^7^Li/^95^Mo-NMR measurements was made at temperature *T* = 10 K with the ^63^Cu free-induction decay (FID) signals from the sample coil. For example, the value of *B*_0_ used for the measurements at the 12 T magnet is determined to be *B*_0_ = 11.9948 T, and at the 14.8 T magnet is *B*_0_ = 14.7427 T. Using the standard Fourier transform (FFT) algorithm in the spectrum analysis, our NMR spectrometer system has a high resolution enabling us to detect a frequency change of 0.01 kHz because of a local electric and/or magnetic field at the atomic scale, as a consequence of CDW, SDW, superconductivity, lattice structure change or any other phase transitions. Noticeably, this is highly sensitive as the honor of the role of an NMR spectrum normally plays. As an example, for a CDW or a charge ordering, it has been experimentally observed that across the transition there is a local field change, which corresponds to a change (spectrum splitting) in NMR frequency to be in the order of ~5–10 kHz, as seen in the blue bronze^29^ Rb_0.3_MoO_3_ and in the TMTSF family^30^, respectively.

## Additional Information

**How to cite this article**: Wu, G. *et al.* Direct observation of charge state in the quasi-one-dimensional conductor Li_0.9_Mo_6_O_17_. *Sci. Rep.* **6**, 20721; doi: 10.1038/srep20721 (2016).

## Supplementary Material

Supplementary Information

## Figures and Tables

**Figure 1:**
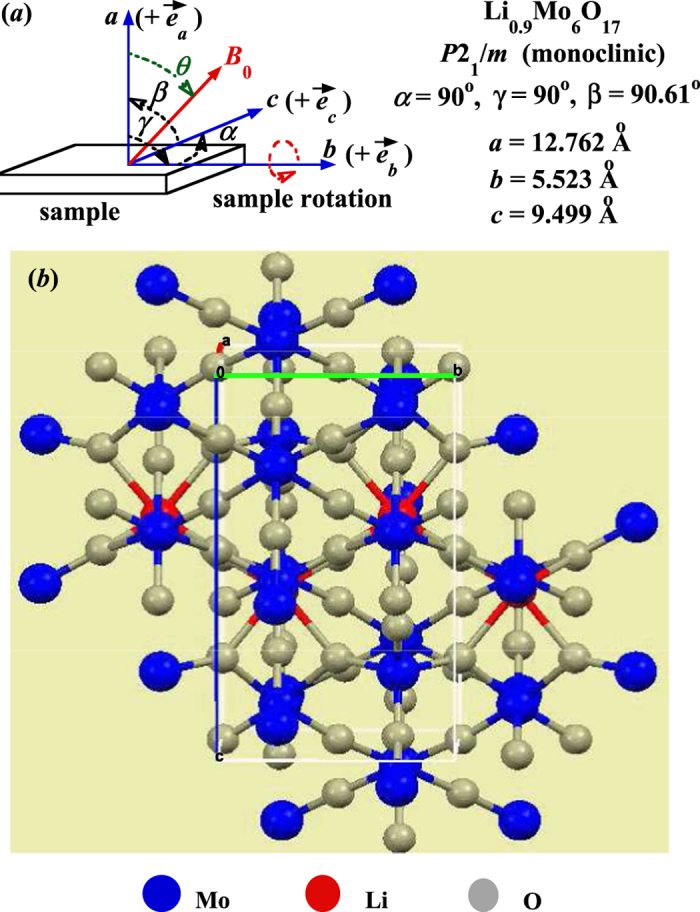
Sample set-up in the NMR experiment and the crystal structure. (**a**) Schematic of the sample rotation around the lattice *b*-axis in the applied magnetic field *B*_0_ (*B*_0_ ⊥ *b*). That the value of the angle *θ* = 0° is for *B*_0_ || *a*, and *θ* is “+” if the sample rotation is clockwise [viewed along the *b*-axis (+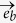)]. Otherwise, *θ* is “−”. For convenience, the values of the lattice constant of Li_0.9_Mo_6_O_17_ [Ref. 34] are also shown on the side. (**b**) The crystal structure of Li_0.9_Mo_6_O_17_ viewed along ~*a* –axis.

**Figure 2:**
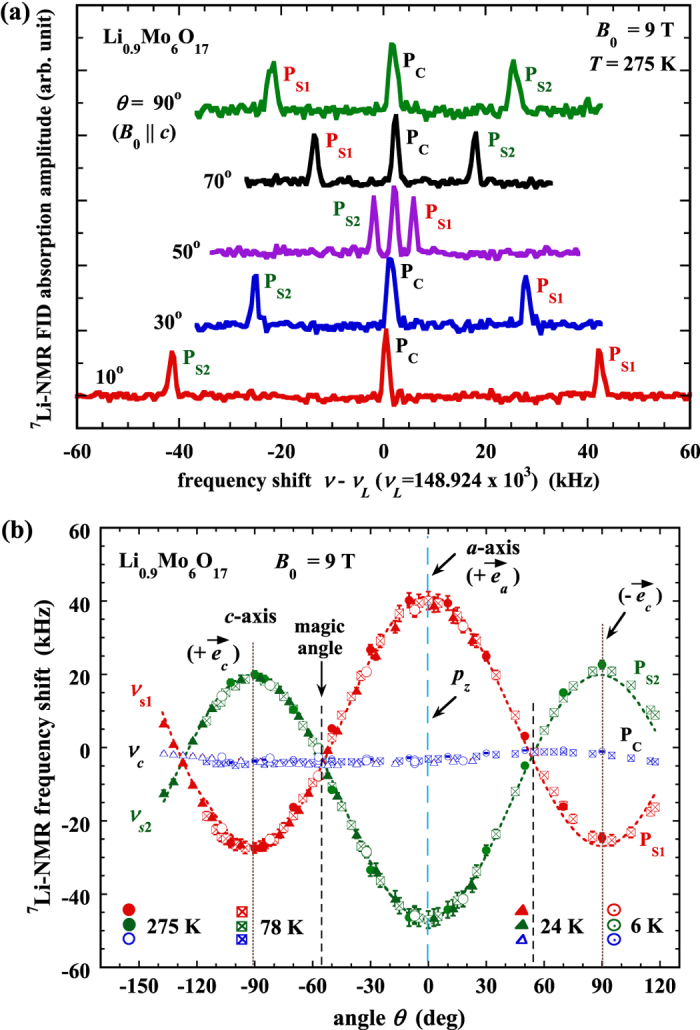
^7^Li-NMR spectrum and frequency shift. (**a**) Angular dependence of the ^7^Li-NMR spectrum of Li_0.9_Mo_6_O_17_, plotted as the ^7^Li-NMR free-induction decay (FID) absorption amplitude versus the NMR frequency shift, at temperature *T* = 275 K with sample rotations around the *b*-axis in the applied magnetic field *B*_0_ = 9 T. (**b**) Angular dependence of the ^7^Li-NMR frequency shift of Li_0.9_Mo_6_O_17_ at various temperatures with sample rotations around the *b*-axis at *B*_0_ = 9 T. The dashed curves are the theoretical fit. The vertical dashed lines are for the directions of the *a*- and *c*-axes, the magic angle, and the principle Z-axis (with quadrupole moment component *p*_z_) of the EFG determined from the experiment.

**Figure 3:**
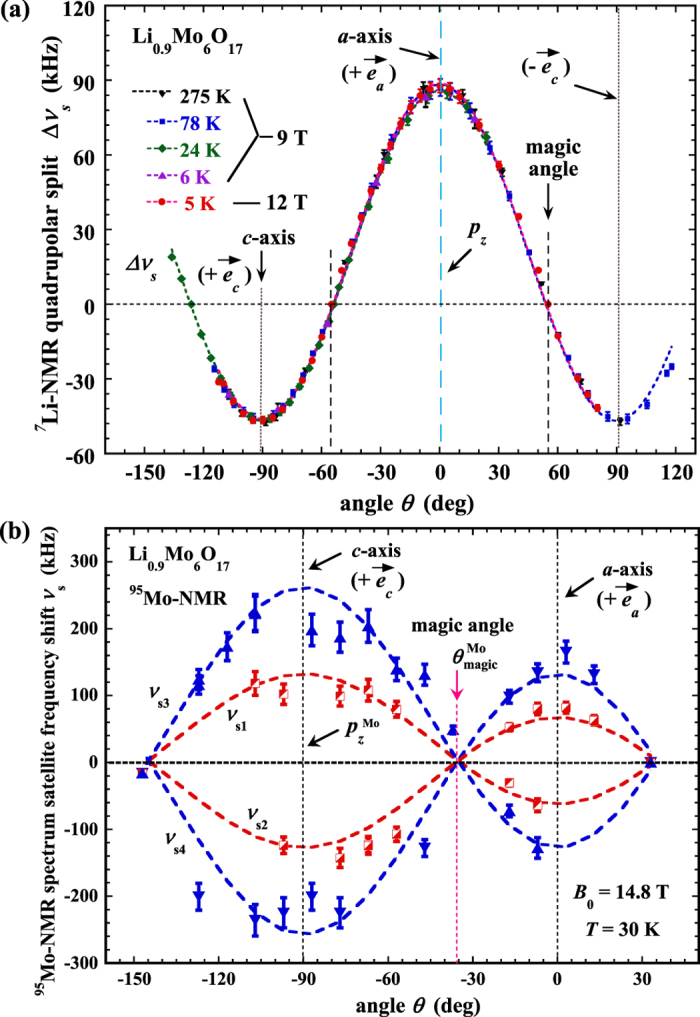
^7^Li-NMR quadrupolar split frequency and ^95^Mo-NMR quadrupolar satellite frequency. (**a**) Temperature and angular dependences of the ^7^Li-NMR quadrupolar split frequency of Li_0.9_Mo_6_O_17_ at the applied magnetic field *B*_0_ = 9 T and 12 T. The dashed curves are the theoretical fit. (**b**) Angular dependence of the ^95^Mo-NMR spectrum satellite frequency at a typical temperature *T* = 30 K with *B*_0_ = 14.8 T. The dashed curves are the theoretical fit with spin quantum *m* − 1/2 = ±1 for the inner satellites which have frequency shifts *ν*_s1_ and *ν*_s2_, and *m* − 1/2 = ±2 for the outer satellites which have frequency shifts ν_s3_ and ν_s4_. The dashed vertical lines indicate the positions of the lattice *a-* and *c*-axes, and the magic angle, as well as the position of the principle Z-axis (*p*_z_) of the EFG at the Mo site obtained from the experiment.

**Figure 4:**
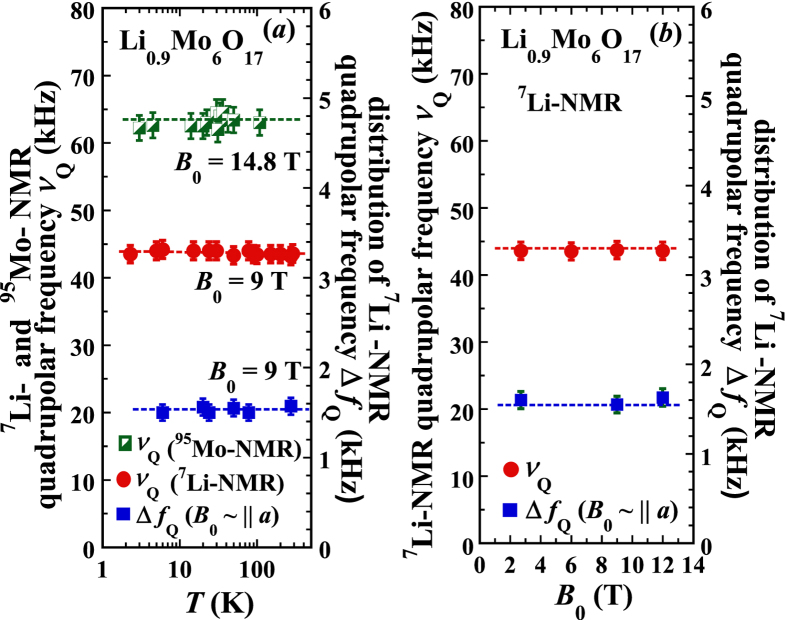
^7^Li- and ^95^Mo-NMR quadrupolar frequency *ν*_Q_ (EFG) and distribution of ν_Q_ (EFG). (**a**) Temperature dependence of the ^7^Li- and ^95^Mo-NMR quadrupolar frequency *ν*_Q_ (EFG) and/or the distribution of it at the applied magnetic field *B*_0_ = 9 T and/or 14.8 T. (**b**) Magnetic field-dependence of the ^7^Li-NMR quadrupolar frequency *ν*_Q_ (EFG) and the distribution of *ν*_Q_ (EFG). The values of Δ*f*_Q_ for the distribution of *ν*_Q_ are obtained from the ^7^Li-NMR spectra at *B*_0_ ~ || *a*, where the total internal magnetic field at the Li site is ~0. The dashed lines are the guides to the eye.
