# Supplementary material for: Direct observation of charge state in the quasi-one-dimensional conductor Li0.9Mo6O17
Source: Sci Rep. 2016 Feb 8;6:20721. doi: 10.1038/srep20721 (PMC4745083; doi:10.1038/srep20721)
Supplement: Supplementary Information [file srep20721-s1.pdf]

Supplementary Information

Direct observation of charge state in the quasi-one-dimensional conductor $Li_{0.9}Mo_6O_{17}$

Guoqing Wu*[1,2,4] , Xiao-shan Ye[1], Xianghua Zeng[ξ1], Bing Wu[3], and W. G. Clark[4]

[1]College of Physics Science and Technology, Yangzhou University,
Yangzhou 225002, China
[2]Department of Physics, University of West Florida,
Pensacola 32514, USA
[3]Department of Math and Computer Science, Fayetteville State University,
Fayetteville, North Carolina 28301, USA
[4]Department of Physics and Astronomy, University of California, Los Angeles,
California 90095, USA

*Email: gwu@physics.ucla.edu [ξ]Email: xhzeng@yzu.edu.cn

A. A brief introduction for the theoretical aspect of the NMR quadrupolar interaction

Generally, an NMR frequency (ν) can be shifted from the Larmor frequency (ν_L) by the quadrupolar interaction of the nuclear quadrupolar moment (Q) with the electric field gradient (EFG) at the probe nucleus. Here the value of ν comes from the NMR experiment, and $\nu_L = \gamma_I \cdot B_0$ (where γ_I is the gyromagnetic ratio of the probe nucleus, and B_0 is the applied magnetic field). γ_I and Q are the constants of the probe nucleus. For example, their values for ^{7}Li are: $^7\gamma_I$ = 16.5471 MHz/T, and ^7Q = – 0.045 barn. Correspondingly, their values for ^{95}Mo are: $^{95}\gamma_I$ = 2.7747 MHz/T, and ^{95}Q = + 0.120 barn (1 barn = 10^{-28} m^2).

An EFG at a probe nucleus comes from the contribution of the effective electron charges at all the lattice sites in the crystal lattice (ligand lattice contribution), plus orbital overlap (contribution of orbital overlaps) and covalent charge contributions (covalence contribution) (Refs. 32, 33). The orbital overlaps are formed by the outer unfilled shell electron orbitals of the probe atom (atom with the probe nucleus) overlapping with those of the nearest neighboring atoms (which are the oxygen 2p orbitals here for both Li and

Mo atoms as determined by the lattice structure of $Li_{0.9}Mo_6O_{17}$), while the covalent charges are caused by the virtual transport of the electrons from the probe atom (which are the Li-2*s* electrons) to the O- 2p orbital shells here.

According to the point-charge model [Refs. 32, 33], to a first approximation, the quadrupolar Hamiltonian H_Q has the simplest expression as

$$H_Q = \frac{eV_{zz}Q}{4I(2I-1)}\left[3I_z^2 - I(I+1) + \frac{1}{2}\eta(I_+^2 + I_-^2)\right], \qquad (1)$$

where e is the absolute value of the electron charge, and η is the EFG asymmetry parameter , $\eta = \left|\frac{V_{xx}-V_{yy}}{V_{zz}}\right|$ $(0 \le \eta \le 1)$. Here V_{xx}, V_{yy}, and V_{zz} are the components of the EFG tensors along the principal X, Y and Z axes, respectively, and they must satisfy: $V_{xx} + V_{yy} + V_{zz} \equiv 0$. I_+, I_- and I_z are the standard nuclear spin quantum operators expressed by the x, y,and z components of the spin I.

In presence of an applied magnetic field B_0, the quadrupolar Hamiltonian H_Q (as a perturbation to the spin Zeeman splitting under the high-field limit), produces a second order quadrupolar frequency shift (ν_C) to the NMR spectrum central line that is often negligible (the first-order part is zero). But its contribution to the NMR spectrum satellites is dominant as it is in the first order, with a qradrupolar frequency shift ν_s (Refs. 32, 33),

$$\nu_s = -\frac{\nu_Q}{2}\left(m - \frac{1}{2}\right)\left(3cos^2\theta' - 1\right) - \eta\frac{\nu_Q}{2}\left(m - \frac{1}{2}\right)sin^2\theta' \cos(2\phi), \qquad (2)$$

where

$$\nu_Q = \frac{3eV_{zz}Q}{2I(2I-1)h} \ . \qquad (3)$$

Here ν_Q is the nuclear quadrupolar frequency, m the spin quantum, and $m - 1/2 = \pm 1, \pm 2, \ldots$ ($|m| \le I$) which are for the transition lines of the NMR spectrum satellites. h is the Planck constant, and θ' and ϕ are the standard spherical angles of B_0 relative to the principal axes X, Y and Z of the EFG (note, $\theta' = 0^o$ is for $B_0 \parallel$ Z).

Interestingly, equation (2) has four parameters, ν_Q, η, θ' and ϕ, all of which can be obtained by the measured angular (θ) dependence of the NMR spectrum satellite frequency shift (ν_s versus θ) with the information of the experimental set-up (for the NMR measurements), even though θ seems to be the only known parameter here (note: θ describes the direction of B_0 relative to the lattice a-axis here in our NMR experiment) (Fig. 1a). These are the parameters describing the internal electric field environment at the probe nucleus, among which the value of ν_Q is a direct measure of the EFG (tensor V_{zz}), as seen from the quantitative relation between ν_Q and the EFG (V_{zz}) in Eq. (3). In fact, this is basically how an NMR experiment can be used to directly observe an electron charge state.

Furthermore, considering that a nuclear quadrupole moment Q (induced in the electron shell of the probe atom) has an antishielding effect [S1] when it interacts with the external point charges in the ligand lattice and with the valence electrons of the probe atom, we have equation (3) to be replaced by a more general form [S2]

$$\nu_Q = (1-\gamma_\infty)\frac{3eV_{zz}Q}{2I(2I-1)h} + (1-R_0)\frac{3eW_{zz}Q}{2I(2I-1)h}\,, \tag{4}$$

where W_{zz} is the principal z-component of the valence EFG. Here γ_∞ and R_0 are the Sternheimer coefficients (antishielding factors) of the probe atom, corresponding to the nuclear quadrupole moment interactions with the external point charges and the valence electrons in the crystal lattice, respectively.

B. Experimental data analysis for the values of the ^{7}Li-/-^{95}Mo NMR quadrupolar frequency ν_Q (EFG) and/or the distribution of ν_Q (EFG)

1. The experimental value of ν_Q (EFG)

With Eq. (2), we can express the ^{7}Li-NMR quadrupolar split ($\Delta\nu_s$) (the frequency difference between the two spectrum satellites) as

$$\Delta\nu_s \equiv \nu_{s1} - \nu_{s2} = \nu_Q\left(3cos^2\theta' - 1\right) + \eta\nu_Q sin^2\theta' \cos(2\phi). \tag{5}$$

Here $m - 1/2 = \pm 1$ is used (for the ^{7}Li-NMR spectrum satellites).

Noticeably, the experimental data shown in Fig. 3a can be well-fitted by Eq. (5). Similar fit using Eq. (2) can also be made for the data shown in Fig. 2b.

Fitted to Eq. (5), for example, with the consideration of the orientations of B_0 relative to the lattice a, b and c axes in our NMR experimental set-up (Fig. 1a), the ^{7}Li-NMR data shown in Fig. 2 gives

$$\nu_Q = 44.0 \pm 0.5 \ \text{(kHz)} ,$$

$$\eta = 0,$$

$$\theta' = \theta, \ \text{and} \ \phi = 0^o.$$

Here that the obtained value $\eta = 0$ can also be seen apparently by the position of the "magic angle", $\theta = \pm 54.7^o$, where $3cos^2\theta - 1 = 0$ is clearly satisfied (note, $\phi = 0^o$ can be determined independently considering the experimental set-up).

This indicates that the lattice a-axis is the principle axis of the Z-component of the EFG, and $V_{xx} = V_{yy}$, i.e., the electric field environment is highly symmetric at the Li site.

Similarly, for ^{95}Mo-NMR, the two inner quadrupolar split $\Delta\nu_s \equiv \nu_{s1} - \nu_{s2}$ for the satellites with frequency shifts ν_{s1} and ν_{s2} also satisfy Eq. (5), while the two outer ones with frequency shifts ν_{s3} and ν_{s4} satisfy

$$\Delta\nu_s \equiv \nu_{s3} - \nu_{s4} = 2\nu_Q\left(3cos^2\theta' - 1\right) + 2\eta\nu_Q sin^2\theta' \cos(2\phi). \qquad (6)$$

Here $m - 1/2 = \pm 2$ is used for the two outer satellites of ^{95}Mo-NMR spectrum (as ^{95}Mo is a spin $I = 5/2$ nucleus).

Fitted to Eq. (6) or Eq. (5), the data shown in Fig, 3b for the ^{95}Mo-NMR quadrupolar split gives

$$\nu_Q \approx 65 \ \text{(kHz)} ,$$

$$\eta = 0,$$

$$\theta' = \theta \pm 90^o, \ \text{and} \ \phi = 0^o.$$

Thus, this indicates that the lattice c-axis is the principle axis of the Z-component of the EFG, and $V_{xx} = V_{yy}$, i.e., the electric field environment at the Mo site is also highly symmetric (on the average).

2. The experimental value for the distribution of ν_Q (EFG)

As described in the text, an NMR spectrum satellite and central line have different origins. The central line is magnetic because it originates from the contribution of the nuclear spin interaction with the electron spins, the applied magnetic field and other local magnetic field sources, while the satellite is electronic (non-magnetic) due to the nuclear quadrupole moment (Q) interaction with the surrounding EFG.

However, an NMR satellite can be both electronically and magnetically broadened [Refs. 32, 33]. The amount of broadening depends on the angle θ which directly reflects the internal electric and/or magnetic field environment (different sources of internal magnetic fields have different angular dependences). Thus an NMR spectrum is extremely sensitive to any change in the electron charge and/or spin state in the lattice at the atomic scale [Ref. 33].

Therefore, in order to evaluate the change in the distribution of charges in the lattice with the ^{7}Li nucleus, for example, the values of Δf_Q (describing the distribution of ν_Q) shown in Fig. 4 are the full-width at half-maximum (FWHM) of the ^{7}Li-NMR spectrum satellite obtained at the direction of $B_0 \sim \parallel a$, where the total internal magnetic field at the Li site is found to be ~ 0 [S3], i.e., at this angle the contribution to the satellite from the local magnetic field sources is minimized. The way to use it for the ^{95}Mo nucleus is similar, but much higher field B_0 is needed due to extreme difficulty of the NMR experiment as described in the Methods section.

C. <u>Theoretical estimation for the values of the ^{7}Li-/-^{95}Mo NMR quadrupolar frequency ν_Q (EFG)</u>

First, in order to theoretically estimate the value of ν_Q (EFG), one needs to have the values of V_{zz}, W_{zz}, γ_∞ and R_0 to be available according to Eq. (4).

Second, considering that the wave function of the Li-2*s* electrons is spherically symmetric (i.e., independent of the spherical coordinates θ and ϕ), we expect the overlap

of the Li-2s electron orbital with the orbitals of O-2p electrons to be negligible. Therefore, here we can focus on the contributions of ligand lattice and the covalence only.

With the point charge model, the point charge contribution (from the ligand lattice) to the EFG can be estimated using [Ref. 32]

$$V_{zz} = \sum_i kq_i \frac{3z_i^2 - r_i^2}{r_i^5} , \qquad (7)$$

where q_i is the effective charges of the ions at each lattice site i, k is the Coulomb constant, and r_i is the distance from the effective charges at the ith lattice site to the probe nucleus.

Based on the lattice structure of $Li_{0.9}Mo_6O_{17}$ [Ref. 29], using Eq. (7) for the ^{7}Li nucleus we theoretically estimated the EFG value V_{zz} = + 8.1279 $\times 10^{20}$ V/m^2.

As for the covalence contribution, the term W_{zz}, it depends on the average value of $\langle\frac{1}{r^3}\rangle$, which is the expectation value of $1/r^3$ over the covalent orbitals [Ref. 32, 33], owing to the virtual electron charge transport from the Li-2s electron shell to the O- 2p orbital shells in ^{7}Li-NMR. This term has a form [Ref. 32, 33]

$$W_{zz} = -e\frac{4k}{3}\langle\frac{1}{r^3}\rangle . \qquad (8)$$

Using the known value $\langle\frac{1}{r^3}\rangle = 0.38\,\text{Å}^{-3}$ [S4], we can obtain the value of $W_{zz} = -7.296 \times 10^{20}$ V/m^2. We also have the known theoretical value of the antishielding factor [S5] $1 - \gamma_\infty$ = 0.75, and we expect $1 - R_0$ = 0.95 for Li^{1+} considering that Li has a very small atomic number. Thus, from Eq. (4) it gives a theoretical value of ν_Q = − 331.0 + 376.3 = 46.3 (kHz), for the ^{7}Li nucleus, which matches well with our measured value ν_Q ≈ 44 kHz .

Similarly, for the ^{95}Mo nucleus we theoretically estimated the EFG value V_{zz} = + 4.762 × 10^{19} V/m^2 on the average from the effective charges at the lattice sites, using Eq. (7). Thus considering that $1 - \gamma_\infty \approx 35.2$ for $Mo^{5.5+}$ [S6], we have the lattice part contribution to ν_Q to be ~ + 714.6 kHz, which is the first term in Eq. (4). In order to theoretically

estimate the valence contribution [the second term in Eq. (4)], one needs to calculate the values of $< 1/r^3 >_{4d}$ and $< 1/r^3 >_{4p}$ needed for the Mo 4*d* and 4*p* orbital overlaps with the O-2p orbitals, respectively.

Since our measured value ν_Q is ~ 65 kHz, we expect this valence contribution to be ~ −649.6 kHz [the second term in Eq. (4)]. This suggests the value of $< 1/r^3 >_{4d} \approx 0.67$ Å^{-3} for the average of $1/r^3$ of the Mo 4*d* orbit by taking $1-R_0 = 0.83$ [S7]. Here the contribution of the Mo 4*p* orbital overlap with O-2*p* can be neglected as it is expected to be much smaller than that of the Mo 4*d*, since much of the Mo 4*p* shell is inside the outer 4*d* shell and it is fully occupied (by the Mo 4*p* electrons) .

Supplementary information references

[S1] Sternheimer, R. On nuclear quadrupole moment, *Phys. Rev*. **80**, 102 (1950).

[S2] Masterov, V. F., Nasredinov, F. S., Seregin, N. P., and Seregin, P. P. Charge states of atoms in the lattices of the high-temperature superconductors $Tl_2Ba_2Ca_{n-1}Cu_nO_{2n+4}$ and $Bi_2Sr_2Ca_{n-1}Cu_nO_{2n+4}$, *J. Exp. Theor.Phys*. **87**, 588 (1998).

[S3] Wu, Guoqing *et al*. Investigation of magnetic dipole field in the quasi-one-dimensional paramagnetic conductor $Li_{0.9}Mo_6O_{17}$, **http://arxiv.org/abs/1410.7793v4** (to be published in *J. Phys.: Condens. Matt.*).

[S4] Barnes, R. G. and Smith, M. V. Electric field gradients of atomic *p* electrons, *Phys. Rev*. **93**, 95 (1954).

[S5] Das, T. P. and Behrsohn, R. Variational approach to the quadrupole polarizability of ions, *Phys. Rev*. **102**, 733 (1956).

[S6] Sternheimer, R. M. Quadrupole antishielding factors of ions, *Phys. Rev*. **159**, 266 (1967).

[S7] Eremin, M. V. and Lavizina, O. V. Unified picture of the distribution of electric field gradient at Cu, O, and T_m sites in $ReBa_2Cu_3O_{7-\delta}$, *JETP* **84**, 80 (1997).
